# Chlorhexidine as the optimal disinfection method for an accidentally dropped autograft during an anterior cruciate ligament reconstruction surgery: A double‐blind study

**DOI:** 10.1002/jeo2.70614

**Published:** 2026-01-11

**Authors:** Soheil Pourheidar, Mehdi Moayedfar, Mahmoud Jabalameli

**Affiliations:** ^1^ Khanevadeh Hospital Isfahan Iran; ^2^ Bone and Joint Reconstruction Research Center, Shafa Orthopedic Hospital Iran University of Medical Sciences Tehran Iran

**Keywords:** ACL drop, autograft, chlorhexidine, contamination, povidone‐iodine

## Abstract

**Purpose:**

No consensus exists on the optimal sterilization method for contaminated grafts during anterior cruciate ligament (ACL) reconstruction surgery. This study evaluated the efficacy of single and multiple sequential disinfectants in sterilizing the contaminated graft.

**Methods:**

Thirty ACLs were harvested from 30 knees during total knee arthroplasty and sectioned into 10 semi‐identical pieces (*n* = 300). All grafts were dropped simultaneously on the operating room floor and left for 60 s. Then, the grafts were assigned to the 10 study groups. (1) The control group used no sterilizing method, and in the other nine groups were used different sterilizing solutions: (2) 1% povidone‐iodine (PI), (3) 4% chlorhexidine (CH), (4) antibiotics including vancomycin and colistin (AB), (5) thrice washings with PI, CH and AB, (6) twice washing with PI and CH, (7) twice washing with PI and AB, (8) twice washing with CH and AB, (9) once washing with normal saline, and (10) thrice washings with normal saline. A wet swab was also rubbed on the operating room floor. The researchers responsible for cultivation and culture analysis were blinded to the graft tags in the various groups.

**Results:**

Statistical comparisons of contamination rates between groups were performed using chi‐square tests for categorical data, with Fisher's exact tests for pairwise comparisons. A *p* value of <0.05 was considered statistically significant for individual comparisons. Twenty‐nine of the 30 control grafts were positive for bacterial culture, primarily for *Staphylococcus epidermidis* (*n* = 28) and *Micrococcus luteus* (*n* = 13). Eight samples in the PI group were positive for bacteria. No bacterium was isolated in the CH group. Grafts washed once and thrice with normal saline were positive for bacteria in 13 and 8 samples, respectively.

**Conclusion:**

This study found the 4% CH solution to be the most effective method for sterilizing autografts accidentally dropped on the operating room floor.

**Level of Evidence:**

NA (cadaveric, animal and basic science studies).

**Abbreviations**
AB: antibiotics
ACL: anterior cruciate ligament
ACLR: anterior cruciate ligament reconstruction
CH: chlorhexidine
NS: normal saline
PI: povidone‐iodine

## INTRODUCTION

One frequently occurring event is an anterior cruciate ligament (ACL) injury, which often happens during sports among young adults [2]. The rate of ACL tears is increasing rapidly due to the growing participation in sports activities within society [5]. ACL reconstruction (ACLR) is frequently used to treat ACL ruptures, with an incidence of almost 51.2 procedures per 100,000 persons per year [11]. Along with the increased incidence of an ACL tear, the number of ACLRs also increases [11]. Therefore, optimizing ACLR results and reducing its morbidities are of critical importance. In rare instances, especially during ligamentous reconstruction of the knee, graft contamination can occur during surgical procedures, and between 0.6% and 1.8% of ACLR operations experience this complication [3]. That mostly happens when the surgeon uses an autograft for ACLR [16]. As tendon grafts are not only used for ACL reconstructions, these study findings can support other graft surgeries. The involved surgeon has specific options in case such an event occurs, including sterilization or cleansing of the contaminated graft, using an allograft, harvesting a new graft from the contralateral or ipsilateral knee, or delaying the graft reconstruction for a later time [13]. It is feasible to encourage tissue graft decontamination during ACL procedures, provided that the method used complies with the recommended protocol and an efficient sterilizing agent is employed [7]. To reduce the risk of complications of re‐surgery, it is better to refrain from performing it. On the other hand, allografts should be avoided to minimize surgical costs and shorten the duration of surgery. Moreover, using another autograft is no longer rational due to excessive reduction in limb strength; therefore, the most logical choice is to find the most effective way to disinfect the contaminated graft. Such contamination is associated with an increased risk of septic arthritis, which is acknowledged as a rare but serious complication of ACLR [9]. Even so, there is no consensus on the most effective sterilization approach for the intraoperatively contaminated grafts [7]. The study results indicate that Chlorhexidine solutions are more effective than povidone‐iodine (PI) solutions as skin disinfectants [4]. Further research is needed to validate this finding regarding grafts. This study aimed to evaluate the efficacy of different decontamination strategies in preventing graft contamination after manually dropping the grafts on the operating room floor and performing various sterilization techniques on them. This study hypothesized that more prolonged disinfectant exposure would result in lower rates of positive microbial cultures.

## MATERIALS AND METHODS

### Patients' selection

**Figure 1:**
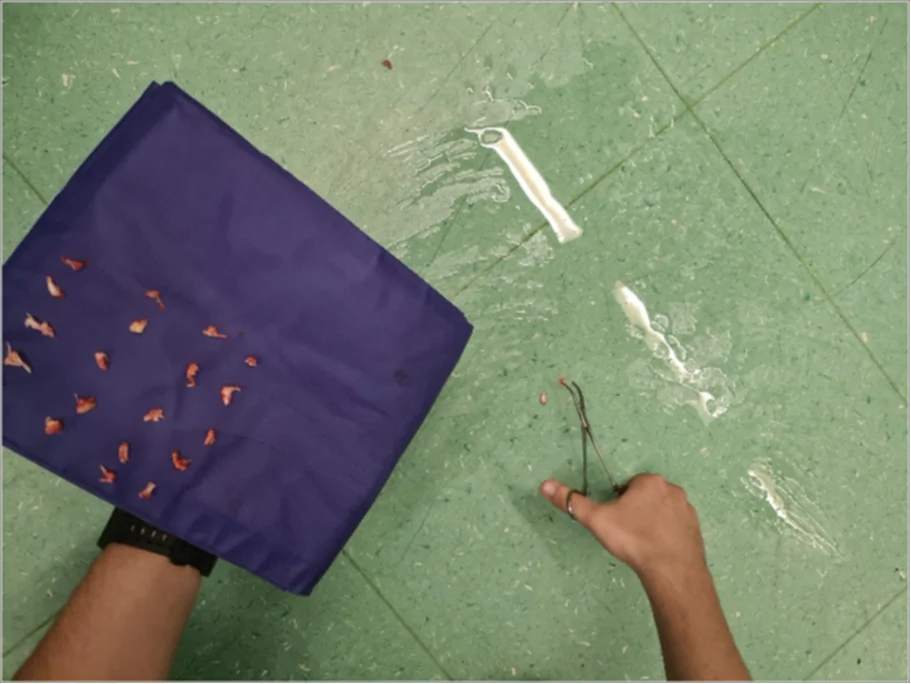
All pieces of the grafts were dropped simultaneously on the operating room floor and left for 60 s.

The ACLs used in this study were harvested under sterile conditions from 30 knees during a primary total knee arthroplasty (TKA). There was only one inclusion criterion: people older than 60 years old. And there is only one exclusion criterion: people who showed post‐surgery infection. A total of 30 knee arthroplasties were performed for osteoarthritis, and in all 30 cases, the ACL was intact. Conversely, none of the patients undergoing total knee arthroplasty developed a postoperative infection. While the surgeon continued the TKA surgery, another person performed the following procedure. sample harvesting and preparation:

The harvested ligaments were then sectioned longitudinally into ten semi‐identical pieces. Following this, all pieces of the grafts were dropped simultaneously on the operating room floor and left for 60 s. Then, by using sterile forceps, the grafts were picked up (Figure 1). At the same time, a culture swab was taken of the floor at the same spot where the graft was dropped. They were immediately transferred to a sterile operating table in another room, where cultures and solutions were placed. Regarding graft handling, sterile devices were used to transfer grafts to the other room in the operating theatre.

Then the grafts were labelled 1–10 and assigned to 1 of the 10 experimental groups, categorized as:

(1) Control Group: where no washing or sterilizing was carried out, (2) Sterilizing with 1% PI solution, (3) 4% Chlorhexidine solution (CH), (4) Antibiotic solution (AB) (vancomycin 10 mg/mL and colistin 10,000 U/mL), (5) Povidone‐iodine solution, chlorhexidine solution and antibiotic solution (PI + CH + AB), (6) Povidone‐iodine and chlorhexidine solutions (PI + CH), (7) Povidone‐iodine solution and antibiotic solution (PI + AB), (8) Chlorhexidine solution and antibiotic solution (CH + AB), (9) washing with normal saline once (One‐NS), and (10) washing with normal saline thrice (Three‐NS).

### Microbiological evaluation

**Figure 2:**
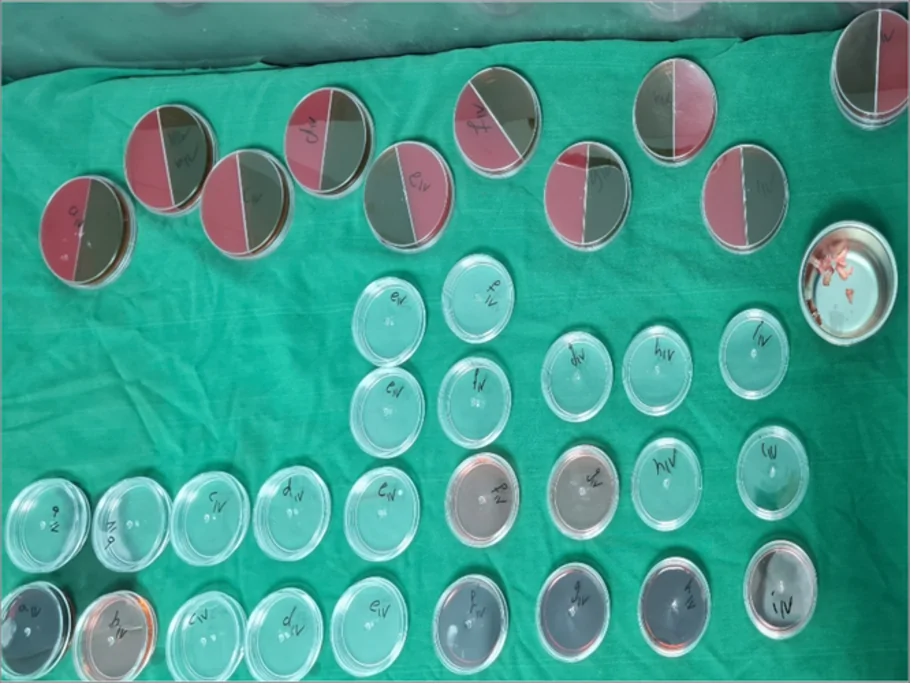
After the contamination, the grafts were placed in the sterilizing or washing solutions for 5 min. In groups where a combination of disinfectants was implemented, the grafts were placed separately in each disinfectant solution for 5 min.

In groups where a combination of disinfectants was implemented, the grafts were placed separately in each disinfectant solution for 5 min. After the contamination, the grafts were placed in the sterilizing or washing solutions for 5 min. In groups where a combination of disinfectants was implemented, the grafts were placed separately in each disinfectant solution for 5 min (Figure 2). Subsequently, the grafts were rubbed with the culture media and sent to the microbiology department for bacteriological examination. A wet swab was also rubbed on the operating room floor, where the grafts were dropped, and cultured to match isolates extracted from grafts. The researchers responsible for cultivation and culture analysis were blinded to the graft tags in various groups, as they were blinded to the meaning of each label (from A to G for each group or category). Moreover, all groups in each series were labelled with numbers 1–30. The environmental conditions were in accordance with the operating room standard: 22–24°, 50%–60% humidity, 20–25 air changes per hour with positive pressure and a HEPA filter.

### Bacteriological examinations

Tissue specimens were inoculated into blood agar and eosin methylene blue agar. Floor swabs were inoculated directly onto culture media. The plates were incubated aerobically at 37°C for 48 h. Laboratory identification of bacteria was performed using Gram staining, colonial morphology and biochemical reactions, including the coagulase test (for gram‐positive organisms), oxidase, catalase reaction, sulphur indole, triple sugar iron, urease, motility, Voges–Proskauer, citrate test and methyl red test (for gram‐negative organisms). The same person reads all cultures. Species were then identified in all positive cultures (more than two colonies were cultured). The senior microbiologist based in the laboratory performed species identification in positive cultures.

### Statistical analysis

Statistical comparisons of contamination rates between groups were performed using chi‐square tests for categorical data, with Fisher's exact tests for pairwise comparisons. These tests are non‐parametric and do not require assumptions of normality. Independence of observations was ensured by design. Bonferroni correction was applied to control for multiple comparisons (Table 1). A *p* value of <0.05 was considered statistically significant for individual comparisons. In contrast, a corrected p‐value threshold of 0.0009 was used after Bonferroni adjustment to control for Type I error in pairwise tests. All statistical analyses were conducted using Python (version 3.13.3) and relevant libraries (Table 2).

**Table 1:** Frequency of contamination, proportion and 95% confidence intervals (CIs).

Group	Positive	Negative	Percentage	95% CI lower	95% CI upper
Wet swabs	27	3	90	0.744	0.965
Control	29	1	96.7	0.833	0.994
PI	8	22	26.7	0.142	0.444
CH	0	30	00.0	0.000	0.114
AB	1	29	3.3	0.006	0.167
One‐NS	13	17	43.3	0.274	0.608
Three‐NS	8	22	26.7	0.142	0.444
PI + CH + AB	0	30	00.0	0.000	0.114
PI + CH	1	29	3.3	0.006	0.167
PI + AB	3	27	10.0	0.035	0.256
CH + AB	1	29	3.3	0.006	0.167

**Table 2:** Pairwise Fisher's exact test *p* values with Bonferroni correction.

Group 1	Group 2	*p*	Significant (after Bonferroni)
Wet swabs	Control	0.6120	No
Wet swabs	PI	<0.0001	Yes
Wet swabs	CH	<0.0001	Yes
Wet swabs	AB	<0.0001	Yes
Wet swabs	One‐NS	0.0003	Yes
Wet swabs	Three‐NS	<0.0001	Yes
Wet swabs	PI + CH + AB	<0.0001	Yes
Wet swabs	PI + CH	<0.0001	Yes
Wet swabs	PI + AB	<0.0001	Yes
Wet swabs	CH + AB	<0.0001	Yes
Control	PI	<0.0001	Yes
Control	CH	<0.0001	Yes
Control	AB	<0.0001	Yes
Control	One‐NS	<0.0001	Yes
Control	Three‐NS	<0.0001	Yes
Control	PI + CH + AB	<0.0001	Yes
Control	PI + CH	<0.0001	Yes
Control	PI + AB	<0.0001	Yes
Control	CH + AB	<0.0001	Yes
CH	One‐NS	<0.0001	Yes
AB	One‐NS	0.0004	Yes
One‐NS	PI + CH + AB	<0.0001	Yes
One‐NS	PI + CH	0.0004	Yes
One‐NS	CH + AB	0.0004	Yes

## RESULTS

According to the assessment of microbial exposure at the donor site, post‐surgery infections were counted, with zero patients. Twenty‐nine of the 30 control grafts were positive for bacterial culture (96.66%). *Staphylococcus epidermidis* (*S. epidermidis*) was the most frequently isolated bacterium in 28 of the 29 positive control grafts (96.55%). *Micrococcus luteus* (*M. luteus*) and *Bacillus subtilis* (*B. subtilis*) were the next most frequently isolated bacteria from 13 and 10 control grafts, respectively (44.82% and 34.48%). *Klebsiella pneumoniae* (*K. pneumoniae*) and *Escherichia coli* (*E. coli*) were the least frequently isolated bacteria from six and three control grafts, respectively (20.68% and 10.34%). The bacteria isolated from the swabs were nearly identical.

In the PI group, eight specimens were positive for bacteria, and the isolated bacteria included *S. epidermidis* (*n* = 6), *M. luteus* (*n* = 2) and *E. coli* (*n* = 1). No bacterium was isolated from the chlorhexidine group. Only one positive culture of *S. epidermidis* was detected in the antibiotic group. With One‐NS sterilization, 13 samples from the graft group were positive for bacteria. The most frequently isolated bacterium in this group was *S. epidermidis* (*n* = 12). Eight samples were positive for bacteria in the graft group with Three‐NS sterilization. Again, the most frequently isolated bacterium in this group was *S. epidermidis* (*n* = 8). A three‐time washing with PI, chlorhexidine and antibiotics led to the complete inhibition of bacterial growth. The two‐time washing of the disinfectants also significantly inhibited bacterial growth. However, PI and antibiotics were less successful than multiple sequential washes containing chlorhexidine (Table 3).

**Table 3:** Number of positive cultures in different types of disinfection methods, *N*.

Group assignment	Wet swabs	Control tissue	PI	CH	AB	One‐NS	Three‐NS	PI + CH + AB	PI + CH	PI + AB	CH + AB
Samples	30	30	30	30	30	30	30	30	30	30	30
Positive samples	27	29	8	0	1	13	8	0	1	3	1
Isolated bacteria											
S. E	13	15	6	0	1	12	8	0	0	2	0
M. L	10	11	2	0	0	5	2	0	1	1	1
B. S	9	9	0	0	0	3	0	0	0	0	0
K. P	6	6	0	0	0	0	0	0	0	0	0
E. C	3	3	1	0	0	3	0	0	0	0	0
Total colonies	1154	661	35	0	1	120	32	0	1	4	1

*Note*: This study simulated graft contamination, which may occur inadvertently during anterior cruciate ligament reconstruction (ACLR). Then, various disinfection methods were examined to decontaminate the grafts and minimize the risk of infection as much as possible. You can see from this table the positive cultures in each group and the total colony numbers of all positive cultures in each group.

The grafts were assigned to 1 of the 10 experimental groups, categorized as: (1) Control Group: where no washing or sterilizing was carried out; (2) Sterilizing with 1% PI solution; (3) 4% Chlorhexidine solution (CH); (4) Antibiotic solution (AB) (vancomycin 10 mg/mL and colistin 10,000 U/mL); (5) PI solution, chlorhexidine solution and antibiotic solution (PI + CH + AB); (6) PI and chlorhexidine solutions (PI + CH); (7) PI solution and antibiotic solution (PI + AB); (8) Chlorhexidine solution and antibiotic solution (CH + AB); (9) One‐time washing with normal saline (One‐NS); and (10) Three‐time washing with normal saline (Three‐NS). After the contamination, the grafts were placed in the sterilizing or washing solutions for 5 min. In groups where a combination of disinfectants was implemented, the grafts were placed separately in each disinfectant solution for 5 min. Subsequently, the grafts were rubbed with the culture media and sent to the microbiology department for bacteriological examination. A wet swab was also rubbed on the operating room floor and cultured to match isolates extracted from grafts. PI: 1% PI solution, CH: 4% Chlorhexidine solution, AB: Antibiotic solution.

**Figure 3:**
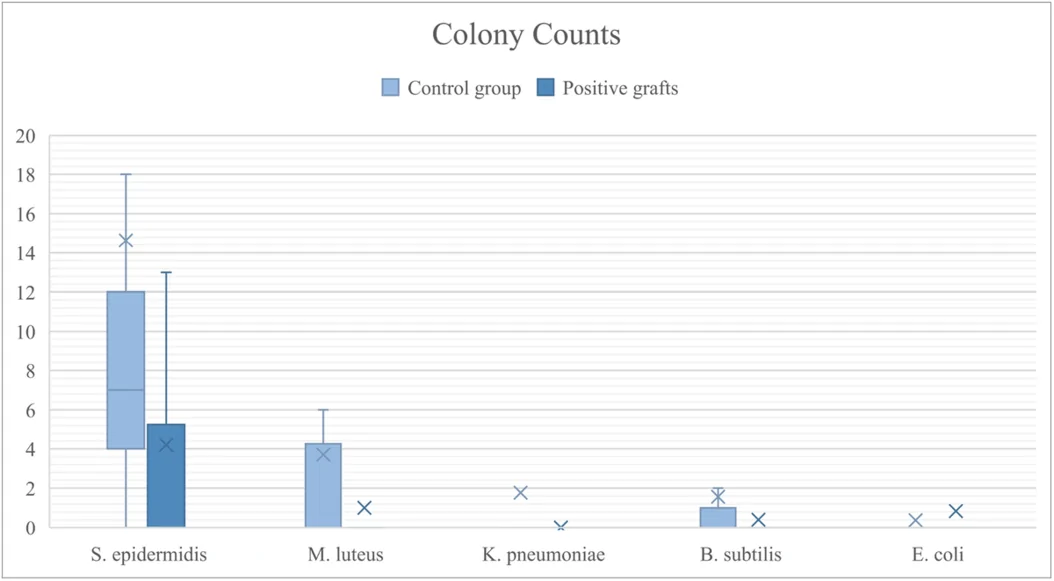
Colony count, *N*.

Following these events, the colony count of each group was enumerated to determine the bacterial load in each group; the mean colony count was 4 for the Three‐NS group and about 9 in the One‐NS group in the positive cultures for bacterial growth. The mean colony number was approximately 4 in the PI group, whereas the mean colony count was approximately 1 in the following groups: AB, PI + CH, PI + ABand CH + AB. Mean colony numbers for *S. epidermidis* and *M. luteus* were 14.63 and 3.7 in the control group and 4.2 and 1 positive cultures, respectively (Figure 3).

The fallen grafts were divided into 10 groups and cultured. This process was repeated 30 times. All cultures were read and counted in a double‐blind manner by the head of the microbiology department of the city laboratory. The type of bacteria and the number of colonies were reported separately, as shown in the graph. The graph indicates that all disinfection methods are effective, as the colony number of positive grafts is lower than that of the control graft. This box and whisker chart includes mean, median, first quartile and third quartile and excludes outliers.

*S. epidermidis*: *Staphylococcus epidermidis*; *M. luteus*: *Micrococcus luteus*; *B. subtilis*: *Bacillus subtilis*; *K. pneumoniae*: *Klebsiella pneumoniae*; and *E. coli*: *Escherichia coli*.

The chi‐square test revealed a statistically significant difference in positive culture rates among the groups (*χ* ^2^ = 187.96, *p* < 0.0001). Post hoc pairwise Fisher's exact tests, with Bonferroni correction (adjusted *p* < 0.0009), demonstrated that the Control and Wet swab groups had significantly higher rates of positive samples compared to all treatment groups (all *p* < 0.0001). Among the treatment groups, chlorhexidine (CH), PI + CH + AB and PI + CH groups had significantly lower positive rates compared to saline washes and PI alone.

## DISCUSSION

This study evaluated the effect of single and multiple disinfecting washing techniques on inhibiting bacterial growth if a harvested graft is accidentally dropped on the operating room floor (simulated contamination). S. E was the primary bacterium isolated from the operating room grafts (29/30 samples). 4% CH solution alone inhibited the growth of all bacteria isolated from the control grafts. The CH combination with PI and AB also inhibited bacterial growth. AB solution was also an effective sterilization solution, with only one positive culture. The bacterium isolated from eight samples disinfected with PI alone showed that a 1% PI solution was not effective in inhibiting bacterial growth. *S. epidermidis* was the primary isolated bacterium in this group as well. Additionally, PI and AB were less successful than combinations containing CH. Three‐NS was associated with a considerably lower colony count than one‐time washing with normal saline.

Plante et al. evaluated methods of graft decontamination in situations when they would accidentally fall on the Each segment was allocated to one of the six study groups (30 segments each): (A) uncontaminated graft (control); (B) graft dropped onto the operating room floor for 5 s; (C) graft dropped onto the operating room floor for 15 s; graft dropped onto the operating room floor for 15 s and then irrigated with normal saline, bacitracin solution, or 4% CH solution for 3 min (groups D, E and F, respectively). Positive cultures were minimal in groups E and F. S. E was the most common isolated bacterium. They concluded that a dropped hamstring autograft could be efficiently sterilized with a bacitracin or 4% CH solution [12]. Bacitracin was not used in the present study. Consistent with the survey of Plante et al., effective sterilization of dropped hamstring autografts was observed using a 4% CH solution. Unlike them, this study was double‐blind and included more groups of disinfection methods, including wet swap, and multiple washings were done for the first time. Consistent with the survey of Plante et al., effective sterilization of dropped hamstring autografts was observed using a 4% CH solution. It is possible that the culture media may not be able to detect contamination well. Therefore, a second control group was used to demonstrate the level of contamination in the environment, and the swab was cultured in each examination.

Burd et al. evaluated the effectiveness of different methods and volumes to decontaminate cadaveric human bone‐tendon allografts inoculated with four different organisms, including *S. epidermidis*, *S. aureus*, *P. aeruginosa* and *K. pneumoniae* [1]. The disinfectants included benzalkonium chloride, castile soap, triple antibiotic, 4% chlorhexidine gluconate and 4% chlorhexidine gluconate plus triple antibiotic. In their study, the only combination that completely sterilized the graft was chlorhexidine powder irrigation and a chlorhexidine/triple antibiotic bath. Moreover, they found that a 2% chlorhexidine solution was as effective as a 4% chlorhexidine solution. They concluded that an effective sterilization method is a 2% chlorhexidine solution for decontaminating human bone‐tendon allografts [1]. In the present study, the efficacy of a 4% chlorhexidine solution was evaluated for sterilizing human tendon grafts. Like the results of Burd et al.'s study, CH irrigation completely sterilized all grafts in the present study. It is pertinent to mention that higher concentrations of CH solution can be toxic to human tissues [6]. If the sterilization effects of 2% chlorhexidine are equivalent to those of 4% concentration, as demonstrated by Burd et al., lower chlorhexidine usage should be considered.

Stanford et al. evaluated the effectiveness of a 10% PI solution in sterilizing contaminated bone‐tendon autografts. Human cadaveric grafts were immersed in either *Staphylococcus aureus* or *Pseudomonas aeruginosa* suspensions [15]. Sterilization was performed with a 10% PI solution for 30 min. Test organisms grew in all six grafts inoculated with *Staphylococcus aureus* after soaking at room temperature and in five out of six after washing at 36°C. Test organisms were grown in five out of six grafts inoculated with *Pseudomonas aeruginosa* after rubbing at room temperature and all six after rubbing at 36°C. They concluded that 10% PI is inadequate for sterilizing a contaminated graft [15]. In this study, PI was used in 1% concentration, which was also ineffective in inhibiting bacterial growth. These observations confirm the inadequacy of the PI solution as a sterilization method for contaminated grafts. Several other studies have also investigated the optimal sterilization method for contaminated grafts [8, 10]. Shen et al., in a systematic review and meta‐analysis, compared the sterilization efficiency of the different disinfectants (4% CH, AB, ratio and 10% PI) on dropped ACL grafts. A total of seven studies met the criteria. The rate of positive cultures was 44.9%, and *Staphylococcus* and *Bacillus* were the most isolated bacteria. The sterilization efficiency of a 4% CH solution was superior to both AB solutions and a 10% PI solution. The sterilization efficiency of the AB solution was superior to that of the 10% PI solution. They concluded that a 4% CH solution is the most reliable sterilization method for decontaminating ACL grafts [14].

The results of the present study, in line with those of earlier investigations, reveal that a 4% CH solution effectively inhibits bacterial growth following accidental contamination of ACL grafts and could be selected as the optimal sterilization method for such contaminations.

The present study had limitations, including the fact that the graft size was smaller than that used for ACLR, since the harvested graft was divided into 10 segments. The size difference could have affected the growth of bacteria. Therefore, it is recommended that future studies be done using actual graft sizes to confirm the results of the present study. In this study, bacitracin or other antiseptics were not used because colistin and vancomycin are easily accessible in every city in this country. The ACLs were not washed in PI, chlorhexidine and antibiotics twice or thrice each because the group number would rise too much. On the other hand, further research is needed to assess the mechanical integrity of the graft after washing. Another important consideration is that this study was conducted in an in vitro setting; a future study should be conducted in an in vivo setting.

## CONCLUSION

Chlorhexidine, at 4%, emerged as the most effective single‐agent decontaminant. However, 1% PI is not an efficient sterilization method for disinfecting grafts. Additionally, more washing times reduce the number of positive cultures and the number of colonies grown. Combination protocols did not significantly outperform it, suggesting that one‐step CH washing may be a time‐ and cost‐efficient strategy in case of graft contamination during ACLR.

## AUTHOR CONTRIBUTIONS

Literature review, manuscript production, data collection and data analysis: Soheil Pourheidar. Study conception, design, data analysis and manuscript review: Mehdi Moayedfar. Study conception, design and manuscript review: Mahmoud Jabalameli. All authors read and approved the final manuscript.

## CONFLICT OF INTEREST STATEMENT

The authors declare no conflicts of interest.

## ETHICS STATEMENT

The ethics statement is not available.

## Data Availability

All raw data are available from the first author on request.
